# Health care claims for primary open-angle glaucoma and retinal vein occlusion from an 11-year nationwide dataset

**DOI:** 10.1038/s41598-017-07890-6

**Published:** 2017-08-14

**Authors:** Hae-Young Lopilly Park, Younhea Jung, Kyungdo Han, Mee Yon Lee, Chan Kee Park

**Affiliations:** 1 Department of Ophthalmology and Visual Science, Seoul St. Mary’s Hospital, College of Medicine, The Catholic University of Korea, Seoul, South Korea; 2 Department of Biostatistics, The Catholic University of Korea, Seoul, South Korea; 3 Department of Ophthalmology and Visual Science, Uijeongbu St. Mary’s Hospital, College of Medicine, The Catholic University of Korea, Seoul, South Korea

## Abstract

To evaluate the risk of retinal vein occlusion (RVO) development after primary open-angle glaucoma (POAG) and the risk of POAG development after RVO, we conducted a nationwide, population-based 11-year longitudinal study. National registry data were collected from the Korean National Health Insurance Research Database, comparing 1 025 340 (~2.2%) subjects who were selected from 46 605 433 Korean residents in 2002. Each sampled patients was tracked until 2013. POAG developed in 0.92% of the RVO group (n = 6 826) and in 0.22% of the comparison group. RVO developed in 0.99% of the POAG group (n = 4 138) and in 0.37% of the comparison group. RVO was associated with an increased risk of POAG development (hazard ratio [HR], 3.25; 95% confidence interval [CI], 2.39–4.42) and POAG was associated with an increased risk of RVO development (HR, 5.05; 95% CI, 3.94–6.47). Comorbidity of systemic hypertension and diabetes mellitus further increased the risk of POAG development in the RVO group (HR, 3.58 and HR, 5.98, respectively). Patients with RVO exhibit a significantly higher risk of POAG development and patients with POAG exhibit a significantly higher risk of RVO development, based on an 11-year follow-up period.

## Introduction

Glaucoma is the leading cause of irreversible blindness worldwide. Retinal vein occlusion (RVO) is the second most frequent retinal vascular disease and also one of the sight-threatening conditions. The association between RVO and glaucoma has been reported in many studies^1–10^. The prevalence of glaucoma is much higher in patients with RVO than in the general population^11^. A prospective observational study showed that intraocular pressure (IOP), glaucoma, and glaucomatous cupping were the only factors related to RVO at the optic disc^11^. However, the epidemiologic characteristics of RVO and primary open-angle glaucoma (POAG) are not fully understood. The Beaver Dam Eye Study reported that increased cup-to-disc ratio was a significant risk factor of RVO^10^. The Ocular Hypertension Treatment Study showed that greater cup-to-disc ratio was associated with the development of RVO in ocular hypertensive participants, with no difference between the observation group and the group that received glaucoma hypotensive medications^12^. However, 5-year incidence of RVO was not associated with glaucoma or ocular hypertension in the Beaver Dam Study^8^, although participants with RVO after 10 years of follow-up were more likely to have definite or probable glaucoma at baseline^10^. In contrast, there are few reports of the development of POAG after RVO. A secondary type of glaucoma, neovascular glaucoma, occurs after the development of central RVO (CRVO). One study reported that only one case (2.5%) of POAG developed after CRVO in 40 patients during a 10-year follow-up^13^.

In this study, we investigated the association between RVO and the prospective risk of POAG development and the association between POAG and the prospective risk of RVO development using a nationwide sample of 1,025,340 representative residents of South Korea in the National Health Insurance Service National Sample Cohort 2002–2013 (NHIS-NSC 2002–2013).

## Results

Total of 6,826 subjects were regarded as new incident cases of RVO and 4,138 subjects were regarded as new incident cases of POAG. The mean visits during this period to ophthalmology clinic were similar between RVO and POAG group (2.20 ± 3.50 and 2.21 ± 3.52 per year, respectively; *P* = 0.560). Patients with death occurred 41060 (9.76%) in the POAG group and 40744 (9.69%) in the RVO group during the study period.

Table 1 displays the characteristics of the study population for the RVO group and the comparison group. The subjects with RVO were more likely to experience POAG (*P* < 0.001), hypertension (*P* < 0.001), diabetes mellitus (*P* < 0.001), and dyslipidemia (*P* < 0.001) compared with the comparison group. Age and sex were different between the two groups.

**Table 1:** Characteristics of the study population comparison group without retinal vein occlusion (RVO; n = 413710) and retinal vein occlusion group (n = 6826).

	Comparison Group No. (%)	RVO Group No. (%)	*P* Value
**Primary open-angle glaucoma**			<0.001
No event	412806 (99.78)	6763 (99.08)	
Event	904 (0.22)	63 (0.92)	
**Age (≥65 yrs)**	91292 (22.07)	2729 (39.98)	<0.001
**Sex (Male)**	197714 (47.79)	3397 (49.77)	0.001
**Hypertension**			<0.001
No	319675 (77.27)	4105 (60.14)	
Yes	94035 (22.73)	2721 (39.86)	
**Diabetes mellitus**			<0.001
No	382188 (92.38)	5462 (80.02)	
Yes	31522 (7.62)	1364 (19.98)	
**Dyslipidemia**			<0.001
No	336251 (81.28)	5671 (83.08)	
Yes	77459 (18.72)	1155 (16.92)	
**Household income relative to the median (0–50%)**	169459 (40.96)	2578 (37.77)	<0.001
**Residential area (Rural)**	223658 (54.06)	3725 (54.57)	0.402

RVO = retinal vein occlusion.

Table 2 show the HR for POAG development in subjects with RVO during the 11-year follow-up period using univariate and multivariate Cox regression models. After adjusting for age, sex, residential area, household income, and comorbidities, including systemic hypertension, diabetes mellitus and dyslipidemia, the subjects with RVO were significantly associated with the prospective development of POAG (HR 2.16, 95% CI, 1.58–2.94) on the basis of multivariate Cox regression analysis of all variables. Comorbidities, such as hypertension (HR 1.91, 95% CI, 1.78–2.05), diabetes mellitus (HR 1.42, 95% CI, 1.30–1.56), and dyslipidemia (HR 1.19, 95% CI, 1.08–1.30), were significantly associated with the development of POAG in subjects with RVO. In terms of sociodemographic characteristics, increasing age, and female gender were significantly associated with the development of POAG in subjects with RVO.

**Table 2:** Univariate and multivariate cox regression analysis for development of primary open angle glaucoma after retinal vein occlusion during an eleven-year follow-up period.

Variable	Univariate Cox			Multivariate Cox		
	HR	95% CI	P Value	HR	95% CI	P Value
**Group**						
Comparison group	1 (ref)			1 (ref)		
RVO group	3.25	2.39–4.42	<0.001	2.16*	1.58–2.94	<0.001
**Hypertension**						
No	1 (ref)			1 (ref)		
Yes	2.48	2.33–2.64	<0.001	1.91	1.78–2.05	<0.001
**Diabetes mellitus**						
No	1 (ref)			1 (ref)		
Yes	1.88	1.73–2.04	<0.001	1.42	1.30–1.56	<0.001
**Dyslipidemia**						
No	1 (ref)			1 (ref)		
Yes	2.16	1.98–2.35	<0.001	1.19	1.08–1.30	<0.001
**Age group (yrs)**						
40–64	1 (ref)			1 (ref)		
≥65	2.14	2.01–2.28	<0.001	1.64	1.54–1.76	<0.001
**Gender**						
Male	1 (ref)			1 (ref)		
Female	1.25	1.17–1.32	<0.001	1.16	1.09–1.23	<0.001
**Household income**						
0–50%	1 (ref)			1 (ref)		
50–100%	1.06	1.10–1.13	0.048	1.09	1.02–1.16	0.009
**Residential area**						
Seoul (metropolitan)	1 (ref)			1 (ref)		
Other	1.01	0.95–1.07	0.796	0.96	0.90–1.02	0.206

RVO = retinal vein occlusion, HR = hazard ratio, CI = confidence intervals. *Adjusted for age, gender, income, residential area, hypertension, diabetes mellitus, dyslipidemia.

Table 3 displays the characteristics of the study population for the POAG group and the comparison group. The subjects with POAG were more likely to develop RVO (*P* < 0.001), hypertension (*P* < 0.001), diabetes mellitus (*P* < 0.001), and dyslipidemia (*P* < 0.001) compared with the comparison group. Age and sex were different between the two groups.

**Table 3:** Characteristics of the study population comparison group without primary open angle glaucoma (n = 416595) and primary open angle glaucoma group (n = 4138).

	Comparison Group No. (%)	POAG Group No. (%)	*P* Value
**RVO**			<0.001
No event	415033 (99.63)	4097 (99.01)	
Event	1562 (0.37)	41 (0.99)	
**Age (≥65 yrs)**	92561 (22.22)	1572 (37.99)	<0.001
**Sex (Male)**	199480 (47.88)	1756 (42.44)	<0.001
**Hypertension**			<0.001
No	321529 (77.18)	2383 (57.59)	
Yes	95066 (22.82)	1755 (42.41)	
**Diabetes mellitus**			<0.001
No	384280 (92.24)	3501 (84.61)	
Yes	32315 (7.76)	637 (15.39)	
**Dyslipidemia**			<0.001
No	379625 (91.13)	3497 (84.51)	
Yes	36970 (8.87)	641 (15.49)	
**Household income relative to the median (0–50%)**	170343 (40.89)	1755 (42.41)	0.048
**Residential area (Rural)**	225255 (54.07)	2246 (54.28)	0.790

POAG = primary open angle glaucoma; RVO = retinal vein occlusion.

Table 4 show the HR for RVO development in subjects with POAG during the 11-year follow-up period using univariate and multivariate Cox regression models. After adjusting for age, sex, residential area, household income, and comorbidities, including systemic hypertension, diabetes mellitus and dyslipidemia, the subjects with POAG were significantly associated with the prospective development of RVO (HR 3.27, 95% CI, 2.55–4.19) on the basis of multivariate Cox regression analysis of all variables. Comorbidities, such as hypertension (HR 1.51, 95% CI, 1.43–1.59), diabetes mellitus (HR 2.06, 95% CI, 1.93–2.20), and dyslipidemia (HR 1.32, 95% CI, 1.23–1.41), were significantly associated with the development of RVO in subjects with glaucoma. In terms of sociodemographic characteristics, increasing age and female gender were significantly associated with the development of RVO in subjects with glaucoma.

**Table 4:** Univariate and multivariate cox regression analysis for development of retinal vein occlusion after glaucoma during an eleven-year follow-up period.

Variable	Univariate Cox			Multivariate Cox		
	HR	95% CI	P Value	HR	95% CI	P Value
**Group**						
Comparison group	1 (ref)			1 (ref)		
Glaucoma group	5.05	3.94–6.47	<0.001	3.27^*^	2.55–4.19	<0.001
**Hypertension**						
No	1 (ref)			1 (ref)		
Yes	2.24	2.13–2.35	<0.001	1.51	1.43–1.59	<0.001
**Diabetes mellitus**						
No	1 (ref)			1 (ref)		
Yes	2.10	1.97–2.23	<0.001	2.06	1.93–2.20	<0.001
**Dyslipidemia**						
No	1 (ref)			1 (ref)		
Yes	2.99	2.82–3.18	<0.001	1.32	1.23–1.41	<0.001
**Age group (yrs)**						
40–64	1 (ref)			1 (ref)		
≥65	2.34	2.23–2.45	<0.001	1.86	1.76–1.96	<0.001
**Gender**						
Male	1 (ref)			1 (ref)		
Female	0.92	0.88–0.97	<0.001	0.86	0.82–0.90	<0.001
**Household income**						
50–100%	1 (ref)			1 (ref)		
0–50%	0.88	0.88–0.97	<0.001	0.86	0.81–0.90	<0.001
**Residential area**						
Seoul (metropolitan)	1 (ref)			1 (ref)		
Other	1.02	0.94–1.07	<0.408	0.98	0.93–1.02	<0.405

HR = hazard ratio, CI = confidence intervals. ^*^Adjusted for age, gender, income, residential area, hypertension, diabetes mellitus, dyslipidemia.

**Figure 1:**
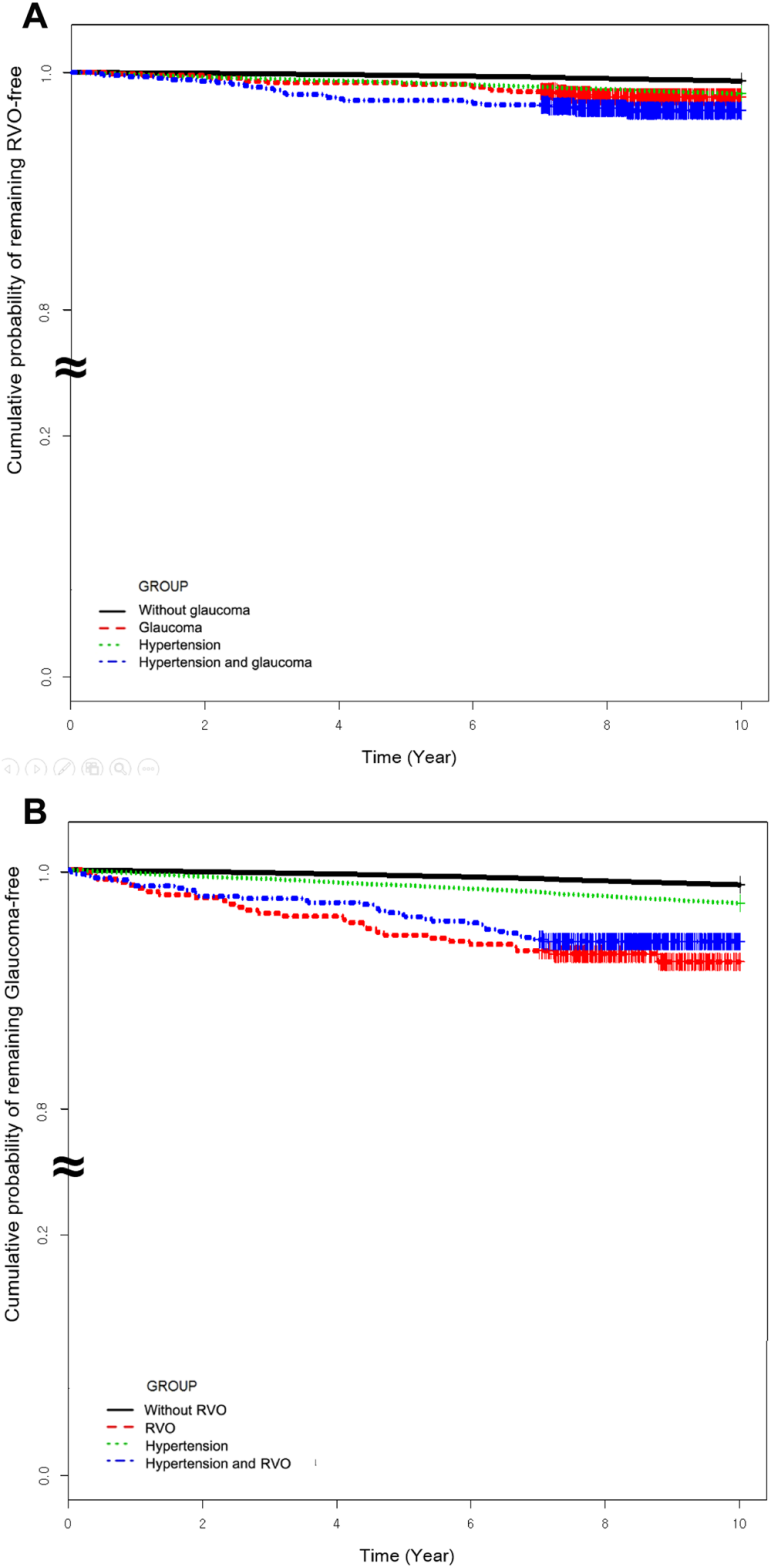
The results of Kaplan-Meier survival analysis. (**A**) Primary open-angle glaucoma (POAG) development in patients with retinal vein occlusion (RVO). (**B**) RVO development in patients with POAG.

Figure 1 shows the results of Kaplan-Meier survival analyses. The log rank tests indicated that the patients with RVO developed POAG significantly more frequently than the comparison group (*P* < 0.001). Comorbidity of hypertension increased the frequency of POAG development in patients with RVO compared to patients with RVO but without hypertension (*P* < 0.001; Fig. 1A). The log rank tests indicated that the patients with POAG developed RVO significantly more frequently than the comparison group (*P* < 0.001). However, comorbidity of hypertension did not increase the frequency of RVO development in patients with POAG compared to patients with POAG but without hypertension (*P* = 0.786; Fig. 1B).

When considering the effect of comorbidities to the development of RVO or POAG, we found different effects according to the baseline disease (Table 5). In patients with RVO who developed POAG, combined comorbidities including hypertension, diabetes mellitus, and dyslipidemia increased the risk of developing POAG. However, in patients with POAG who developed RVO, systemic comorbidities did not increase the risk of developing RVO.

**Table 5:** Multivariate cox regression analysis for development of primary open angle glaucoma or retinal vein occlusion in subjects with and without the baseline diseases (either retinal vein occlusion or glaucoma, respectively).

Conditions	RVO → Glaucoma			Glaucoma → RVO		
	Model 1	Model 2	Model 3	Model 1	Model 2	Model 3
	HR (95% CI)	HR (95% CI)	HR (95% CI)	HR (95% CI)	HR (95% CI)	HR (95% CI)
Baseline disease	3.38 (2.07–5.53)	2.80 (1.71–4.58)	2.89 (1.76–4.72)	7.34 (5.07–10.65)	5.93 (4.09–8.59)	5.93 (4.09–8.59)
Hypertension	2.48 (2.33–2.63)	1.93 (1.80–2.06)	1.93 (1.80–2.76)	2.24 (2.13–2.35)	1.69 (1.61–1.78)	1.50 (1.42–1.59)
Baseline disease + Hypertension	5.31 (3.58–7.89)	3.82 (2.57–5.67)	3.58 (2.41–5.33)	5.92 (4.24–8.25)	4.27 (3.06–5.95)	3.59 (2.57–5.03)
Baseline disease	4.81 (3.55–6.51)	3.82 (2.82–5.18)	3.48 (2.57–4.71)	3.59 (2.51–5.15)	2.83 (1.98–4.07)	2.71 (1.89–3.88)
DM	2.98 (2.91–3.17)	2.41 (2.27–2.56)	2.07 (1.4–2.20)	2.16 (1.98–2.35)	1.78 (1.63–1.94)	1.44 (1.32–1.58)
Baseline disease + DM	10.13 (6.61–15.53)	7.24 (4.72–11.12)	5.98 (3.89–9.19)	3.01 (1.88–6.15)	2.52 (1.39–4.56)	1.99 (1.10–3.61)
Baseline disease	5.28 (3.96–7.04)	4.13 (3.09–5.49)	3.68 (2.76–4.91)	3.77 (2.69–5.23)	2.91 (2.08–4.06)	2.61 (1.87–3.65)
Dyslipidemia	2.09 (1.96–2.23)	1.82 (1.71–1.94)	1.32 (1.23–1.42)	1.88 (1.73–2.05)	1.63 (1.49–1.78)	1.20 (1.09–1.32)
Baseline disease + Dyslipidemia	6.46 (3.95–10.55)	4.85 (2.97–7.93)	3.22 (1.97–5.27)	2.49 (1.12–5.55)	1.89 (0.85–4.23)^*^	1.27 (0.57–2.84)^*^

Model 1: not adjusted.

Model 2: adjusted for age and gender.

Model 3: adjusted for age, gender, income, residential area.

All P values < 0.001 except for^*^.

## Discussion

Our study found that patients with RVO had a higher prospective risk for POAG development during an 11-year follow-up period after adjusting for hypertension, diabetes, and dyslipidemia. In addition, patients with POAG had a higher prospective risk for RVO development during an 11-year follow-up period after adjusting for hypertension, diabetes, and dyslipidemia. These findings suggest that POAG and RVO may serve as risk factors for each other in an interactive manner. In addition, our results confirmed that increasing age, hypertension, diabetes mellitus, and dyslipidemia are associated with POAG development in patients with RVO or with RVO development in patients with POAG. Especially, comorbidities such as systemic hypertension, diabetes, and dyslipidemia were additive risk factors in the development of POAG in patients with RVO, but was not additive in the development of RVO in patients with POAG. It is possible that POAG after RVO may have been detected by chance because patients usually come to the hospital with early symptoms of RVO, but not with early-stage POAG. Therefore, the increased risk of POAG after RVO may be weakened by this limitation. The increased risk of RVO after POAG may be important since the majority of glaucoma cases in Korea are within normal range IOP^14^.

It has been hypothesized that elevated IOP may compress blood vessels and induce subsequent intimal proliferation, leading to collapse of retinal vessel walls. In the OHTS, 23 participants had RVO during a mean follow-up of 9.1 years for a cumulative incidence of 1.4%^12^. The Beaver Dam Eye Study found a 5-year incidence of RVO of 0.8% and the Blue Mountains Eye Study found a 10-year cumulative incidence of 1.6%^8, 9^. In the present study the cumulative incidence of RVO development was 41 (0.99%) among 4,138 glaucoma participants over an 11-year period. However, the Blue Mountains Eye Study found no association between IOP and the incidence of RVO^9^. The Ocular Hypertension Treatment Study (OHTS) reported that the incidence of RVO in patients with ocular hypertension was different according to the RVO subtype, with that of CRVO more than 4 times greater than that of BRVO. Finally, branched RVO (BRVO) has also been reported in eyes with normal-tension glaucoma, suggesting that risk factors other than IOP may contribute to the development of BRVO in eyes with glaucoma^15^. One possible hypothesis is that optic disc cupping due to glaucoma may distort retinal vessels at the disc, which could predispose the veins to occlude resulting in RVO. Beaumont *et al*. reported that a cup-to-disc ratio >0.7 contributed to BRVO in their study population^11^. The Beaver Dam Eye Study and the Ocular Hypertension Treatment Study both reported that high cup-to-disc ratio contributed to RVO development in glaucoma patients with HR of 2.4^8^ and 1.35^12^, respectively. Our previous study showed that changes in the optic disc or retinal nerve fiber layer due to glaucomatous structural changes result in hemodynamic changes at that location, resulting in blood flow stasis by angiography, which may subsequently contribute to endothelial damage or thrombosis formation and RVO occurrence^16^. This could be the reason for our finding of a stronger risk of RVO development after POAG than of RVO contributing to the development of POAG. POAG displayed an approximately 3.3-fold HR for RVO development, compared with a 5.1-fold HR for RVO to develop POAG. Systemic comorbidities did not add to the risk of RVO development in POAG patients, suggesting that structural changes of glaucoma may more strongly contribute to RVO development.

POAG development after RVO has not been studied previously. As mentioned above, it is possible that POAG may have been detected by chance after RVO because patients usually come to hospital with early symptoms of RVO. Participants with asymptomatic POAG might have been included before the RVO development. However, we allowed a 2-year window of time and there was a cumulative increase in the incidence of POAG after RVO. Also, when patients present with RVO symptoms, dilated fundus examination is usually performed and patients with a suspicious optic disc may be referred to glaucoma examination. The development of POAG after RVO was significantly affected by the comorbidity of systemic hypertension. Systemic diseases, such as systemic hypertension and diabetes mellitus with vascular pathophysiologies, have been suggested as possible mechanisms of damage in both glaucoma and RVO. In the present study systemic hypertension, diabetes mellitus, and dyslipidemia were associated with POAG development in patients with RVO and with RVO development in patients with POAG. Vascular dysregulation and autoregulatory dysfunction caused by these systemic diseases may result in both glaucomatous optic neuropathy and RVO^17^. Ocular blood flow was found to be significantly lower in both BRVO eyes and unaffected fellow eyes^18^. RNFL thinning was observed in the fellow eyes of patients with BRVO. It is therefore possible that vascular abnormalities may contribute to the development of POAG in patients with RVO. In particular, hemodynamic changes at the arteriovenous crossing site have been suggested to be important in the occurrence of BRVO, predisposing the eye to endothelial damage and thrombus formation^19, 20^. Systemic diseases related to RVO are also thought to contribute to hemodynamic changes, endothelial damage, and hypercoagulability that may contribute to thrombus formation leading to vessel occlusion^21^. Especially, systemic hypertension, which can contribute to both hemodynamic changes and endothelial damage, add additional risk and may have a role in the development of POAG in RVO patients.

A strength of our study is that the KNHIS is a large population-based database that contains data from a longitudinal cohort and is thus an excellent resource. However, as this study used an insurance database, there are some inevitable limitations. The most important limitation is that the diagnoses of RVO, POAG, and other comorbidities were defined on the basis of ICD-10 codes, which may be inaccurate compared with diagnoses obtained from a medical chart. This data can only provide information about a subject, not information based on ocular level. However, the KNHIS data have been validated by reference to the rates of prevalence of 20 major diseases. Second, the KNHIS is a medical claim-based database, and glaucoma may have been more underdiagnosed than RVO because many POAG patients are asymptomatic in the early stages of disease. Third, we chose medical claim-based controls, who were more likely to have comorbidities compared with controls based on the general population who neither received medical care nor had a specific diagnosis. Thus, the nature of KNHIS database has inherited selection bias. Also, new cases identified at baseline may have included undiagnosed cases which previously started as a chronic disease before 2002. There can be mixed effect of chronic and newly developed glaucoma. We tried to exclude these cases by excluding newly diagnosed cases between 2002 and 2005, and then analyzing newly diagnosed cases starting from 2006, giving a one year lag period. Although this should be considered when interpreting our findings, it may allow a sufficiently powered analysis since it has large sample size. Additionally, the distinguishing features of the Korean medical system, such as the low medical expenses since the government manages universal, single-payer healthcare, and its high health service accessibility, may reduce the possibility of exclusion and underestimation of patients who did not visit a hospital.

In conclusion, patients with RVO exhibited a significantly higher risk of POAG and patients with POAG exhibited a significantly higher risk of RVO, based on an 11-year follow-up period after adjusting for hypertension, diabetes, and dyslipidemia. POAG displayed an approximately 5.1-fold HR for RVO development, compared with a 3.3-fold HR for RVO to develop POAG. It is important for ophthalmologists to examine each patient with RVO or POAG for the development of the other disease during follow-ups.

## Methods

### Statement of Ethics

This study adhered to the tenets of the Declaration of Helsinki, and the NHIS-NSC 2002–2013 project was approved by the Institutional Review Board of the Korean National Health Insurance Service (KNHIS). This study design was reviewed and approved by the Institutional Review Board of St. Mary’s Hospital, Seoul, South Korea. The need for written informed consent was waived by our Review Board.

### Database

In Korea, all nationals are obligated to enroll in the KNHIS. This includes Korean National Health Insurance scheme, which covers 97% of the population in the country. The remaining 3% of population are covered by the Medical Assistance Program and the Medical Care for Patriots and Veterans Affairs Scheme. Thus, nearly all of the data in the health system are centralized in large databases. In Korea, patients with KNHIS pay for 30% of their total medical expenses when using medical facilities, and medical providers receive the other 70% from the KNHIS, for which they must submit claims. Claims include data regarding diagnostic codes, procedures, prescription drugs, personal information about the patient, information about the hospital, the direct medical costs of both inpatient and outpatient care, dental services, and demographic information. In these data sets each patient is identified by a 13-digit social identification number that is given at birth therefore no healthcare records of the patients were duplicated or omitted. Further, diagnostic codes used by the KNHIS are based on the international classification of disease, 10^th^ revision (ICD-10), and are submitted directly by the patient’s doctor.

### Study Sample

We used KNHIS data recorded from 2002 through 2013, which were released in 2015. The data set consists of 1,025,340 nationally representative subjects (~2.2% of the total population), sampled from the entire population in the KNHIS in 2002. The data were produced by the KNHIS using a systemic sampling method to generate a representative sample of the total 46,605,433 Korean residents in 2002. This database includes all medical claims filed from January 2002 to December 2013.

A RVO group and a comparison group without RVO were generated. The RVO group included all patients who received inpatient and outpatient care between January 2003 and December 2005 (index date) for an initial diagnosis of RVO (ICD-10 code H34.8, corresponding to CRVO or venous tributary [branch] occlusion). A POAG group and a comparison group without glaucoma were generated. The POAG group included all patients who received glaucoma medications during the whole study period with at least two visits for either inpatient or outpatient care for POAG (ICD-10 code H401) between January 2003 and December 2005. We excluded subjects who had been treated for RVO and POAG in 2002 (n = 1,139) to exclude patients with chronic conditions and to ensure that only subjects with new episodes were included. For the same reason, patients who had a diagnosis of POAG before the diagnosis of RVO before the index date (n = 35) were excluded from analysis of the prospective risk of POAG development among the RVO group and patients who had a diagnosis of RVO before the diagnosis of POAG before the index date (n = 21) were excluded from the analysis of the prospective risk of RVO development among the POAG group. We included only patients over the age of 40. Then, we added a one year lag period to ensure only newly diagnosed cases were identified as disease development. Since glaucoma is an asymptomatic disease, patients who undergo ocular examination due to RVO could have been newly diagnosed as glaucoma even though the disease condition started before the index date. Analysis of the identification of incident cases started at 2006. All remaining cases had a disease-free period of at least 2 to 5 years before the earliest claim related to the second disease diagnostic code. Finally, 420,733 eligible subjects between 2003 and 2005 were identified after excluding potential preexisting cases of RVO and 420,536 eligible subjects for POAG analyses. Among these cases, 6,826 subjects were regarded as new incident cases of RVO and 4,138 subjects were regarded as new incident cases of POAG. Each patient was tracked based on his or her index dates of ambulatory and inpatient care visits during the 11 years from 2003 to 2013 to detect development of RVO or POAG. Death during the study period were handled as dropouts and were to be censored values.

### Independent Variables

The regression models were adjusted for patient age (40–64, or >65 years), sex, household income (<50% or 50–100% of the median), and geographic location according to two regions (Seoul, or other areas). Korean citizens who are covered under KNHIS are categorized as insured employees, insured self-employed individuals, and medical aid beneficiaries. Low-income individuals (<50% of the median) were defined as insured employees or insured self-employed individuals in the first to fifth income deciles, and medical aid beneficiaries. High-income individuals were defined as insured employees or insured self-employed individuals in the fifth to tenth income deciles. Geographic location was classified as Seoul (South Korea’s metropolitan capital city) and other areas (including the largest South Korean province, the second largest metropolitan city, two of the adjacent second-largest provinces, and all areas other than the previously mentioned subdivisions). Comorbidities, such as hypertension (ICD-10 code I10, I11, I12, I13, and I15), diabetes mellitus (ICD-10 code E11-E14 and prescription of antidiabetic medications, as defined by the Korean Diabetes Association), and dyslipidemia (ICD-10 code E78) may be associated with an increased risk of both diseases. All these baseline variables were measured at baseline, which is 2002.

### Statistical Analysis

Descriptive statistics of the study population are presented, and chi square tests were performed to examine the differences between the two groups. To identify the risk of POAG or RVO development, we calculated hazard ratios (HRs) with 95% confidence intervals (CIs) and analyzed these data using a Cox proportional hazard regression model. In the RVO group, adjusted HR of prospective POAG development after adjusting for confounding comorbidities (hypertension, diabetes mellitus, and dyslipidemia) and sociodemographic characteristics (age, gender, income, and geographic location) was calculated. In the POAG group, adjusted HR of prospective RVO development after adjusting for confounding comorbidities (hypertension, diabetes mellitus, and dyslipidemia) and sociodemographic characteristics (age, gender, income, and geographic location) was calculated. Subjects without the condition served as the referent group. We analyzed association of each disease development to find out the effect of comorbidities using three models. Model 1 did not adjust any confounders. Model 2 adjusted age and gender. Model 3 additionally included other confounders: income, and geographic location. The overall cumulative incidence was calculated using the Kaplan-Meier curves for the 11-year follow-up period, and the log-rank test was performed to examine the differences between groups. A significance level of 0.05 was selected. The statistical package SAS System for Windows, version 9.3 software, was used for analyses.
